# Evaluation of Topical and Subconjunctival Injection of Hyaluronic Acid-Coated Nanoparticles for Drug Delivery to Posterior Eye

**DOI:** 10.3390/pharmaceutics14061253

**Published:** 2022-06-13

**Authors:** Cheng-Han Tsai, Le Ngoc Hoang, Chun Che Lin, Liang-Chen Pan, Chiao-Li Wu, I-Chan Lin, Peng-Yuan Wang, Ching-Li Tseng

**Affiliations:** 1 Graduate Institute of Biomedical Materials and Tissue Engineering, College of Biomedical Engineering, Taipei Medical University, No. 250, Wu-Hsing Street, Taipei 11031, Taiwan; joyo4180@gmail.com (C.-H.T.); lengochoang252@gmail.com (L.N.H.); 2 Institute of Organic and Polymeric Materials and Research and Development Center for Smart Textile Technology, National Taipei University of Technology, Taipei 106344, Taiwan; cclin0530@mail.ntut.edu.tw; 3 School of Biomedical Engineering, College of Biomedical Engineering, Taipei Medical University, No. 250, Wu-Hsing Street, Taipei 11031, Taiwan; b812107043@tmu.edu.tw (L.-C.P.); b812107032@tmu.edu.tw (C.-L.W.); 4 Department of Ophthalmology, Shuang Ho Hospital, Taipei Medical University, New Taipei City 23561, Taiwan; ichanlin@gmail.com; 5 Department of Ophthalmology, School of Medicine, College of Medicine, Taipei Medical University, Taipei 11031, Taiwan; 6 Oujiang Laboratory, Key Laboratory of Alzheimer’s Disease of Zhejiang Province, Institute of Aging, Wenzhou Medical University, Wenzhou 325000, China; 7 International Ph.D. Program in Biomedical Engineering, College of Biomedical Engineering, Taipei Medical University, Taipei 11031, Taiwan

**Keywords:** posterior eye, hyaluronic acid, surface charges, nanoparticles, drug delivery, eyedrops, subconjunctival injection, retina

## Abstract

Posterior eye diseases, such as age-related macular degeneration and diabetic retinopathy, are difficult to treat due to ineffective drug delivery to affected areas. Intravitreal injection is the primary method for posterior eye drug delivery; however, it is usually accompanied by complications. Therefore, an effective and non-invasive method is required. Self-assembling nanoparticles (NPs) made from gelatin-epigallocatechin gallate (EGCG) were synthesized (GE) and surface-decorated with hyaluronic acid (HA) for drug delivery to the retinal/choroidal area. Different HA concentrations were used to prepare NPs with negative (GEH−) or positive (GEH+) surface charges. The size/zeta potential and morphology of the NPs were characterized by a dynamic light scattering (DLS) system and transmission electron microscope (TEM). The size/zeta potential of GEH+ NPs was 253.4 nm and 9.2 mV. The GEH− NPs were 390.0 nm and −35.9 mV, respectively. The cytotoxicity was tested by adult human retinal pigment epithelial cells (ARPE-19), with the results revealing that variant NPs were non-toxicity at 0.2–50 µg/mL of EGCG, and that the highest amount of GEH+ NPs was accumulated in cells examined by flowcytometry. Topical delivery (eye drops) and subconjunctival injection (SCI) methods were used to evaluate the efficiency of NP delivery to the posterior eyes in a mouse model. Whole eyeball cryosections were used to trace the location of fluorescent NPs in the eyes. The area of fluorescent signal obtained in the posterior eyes treated with GEH+ NPs in both methods (eye drops: 6.89% and SCI: 14.55%) was the greatest when compared with other groups, especially higher than free dye solution (2.79%). In summary, GEH+ NPs can be transported to the retina by eye drops and SCI; in particular, eye drops are a noninvasive method. Furthermore, GEH+ NPs, characterized by a positive surface and HA decoration, could facilitate drug delivery to the posterior eye as a useful drug carrier.

## 1. Introduction

Variant ocular diseases in a large population are common occurrences worldwide, with Koichi Ono et al. reporting the existence of 314 million people with visual impairments globally, of whom 269 million people have low vision capabilities, with the remaining 45 million people rendered blind [1]. Most eye diseases involve three main regions: the anterior segment [2,3,4], posterior segment [5,6,7], and intraocular part [8]. In particular, posterior eye disease (age-related macular degeneration [AMD]) and intraocular problems (endophthalmitis) are very difficult to treat due to their anatomical location and biological protective mechanisms, such as the presence of the blood retinal barrier (BRB) [9] and the layer of retinal pigment epithelium (which slows the penetration of drugs from the bloodstream to the back of the eye) [9,10]. Therefore, the use of high-dose drugs to increase the effectiveness of retinal disease treatment is required, but this increases the risk of side effects [11]. Recently, alternative ocular drug delivery systems or administration routes have been increasingly applied [12,13]. There are several methods of ocular drug delivery to the retinal area, including topical administration (eye drops), subconjunctival injections, subretinal injections, and intravitreal injections (IVTs). However, intraocular injections, such as IVTs, are an invasive method that can cause undesirable problems, such as endophthalmitis, hemorrhage, cataracts, and retinal detachment [14,15]. Drug transportation from the systemic circulation via oral medication is another avenue for treatment, but it suffers from very low levels of efficiency in the retina area [9,16].

To improve the efficacy of drug administration to the posterior eye segment accompanied by reduced side effects, many new drugs with special formulations have been introduced in efforts to target delivery via modified methods and routes [9,13,17,18]. Current developments in nanoparticles as drug carriers have become a strategy to enhance drug retention/permeation in the eye for delivery to the target area [19,20], as well as via a prolonged drug release profile in the posterior ocular tissue through conventional eye drops or other least-invasive injection methods [17,21]. The development of biodegradable polymeric nanoparticles, such as liposomes, poly lactic-co-glycolic acid (PLGA), chitosan, gelatin, and hyaluronic acid (HA), for ocular drug delivery and the enhancement of the therapeutic effect in the eye has been studied [9,19,22,23,24]. Among these, gelatin is one of the most prominent materials and has been recognized as safe by the FDA because of its biodegradability, biocompatibility, non-antigenicity, low cost, and ease of availability [25,26].

In our previous study, gelatin-epigallocatechin gallate (EGCG) self-assembling nanoparticles (GE) with HA coating on the surface (GEH) were designed and synthesized for dry eye treatment [22]; as such, GEH NPs were used in this study. HA is a natural polysaccharide that can bind to the CD44 receptor on retinal pigment epithelial (RPE) cells as a guiding motif to the retina [23]. The surface charge of GEH (+/−) was modulated by adjusting the amount of HA added so as to study the charge and targeting effect of the nanoparticles for drug delivery to the posterior eye. 

**Figure 1:**
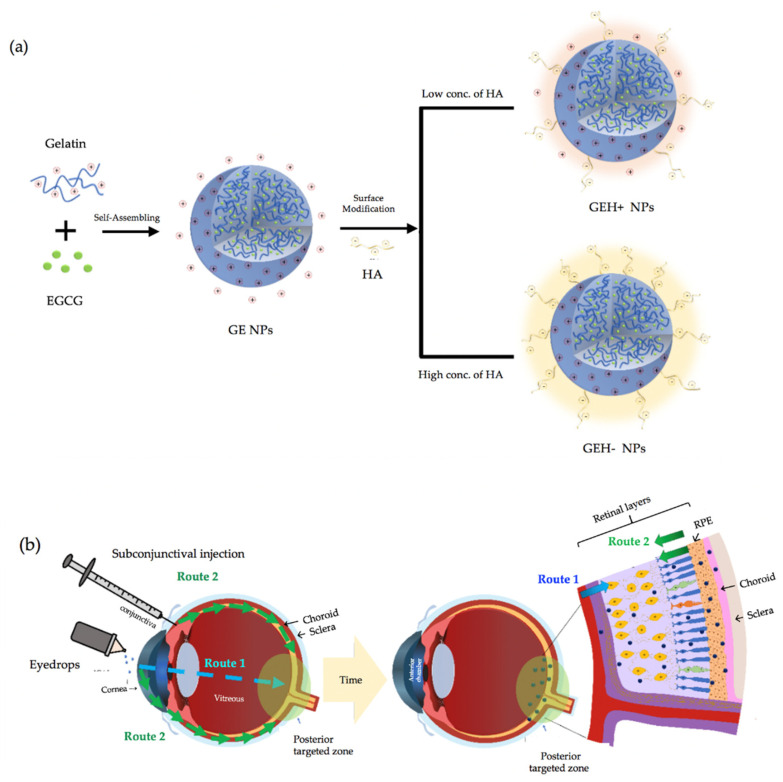
Schematic drawing of (**a**) self-assembled GE NPs with different HA addition forming cationic/ionic surface, and (**b**) two methods (eyedrops and subconjunctival injection) which were used to evaluate delivery of nanoparticles to the posterior eye segment. (Route 1: trans-corneal route, Route 2: non-corneal route).

The aim of this study was to evaluate biopolymeric nanoparticles, gelatin/EGCG self-assembling NPs, with or without HA modification on the surface (Figure 1a), to influence its delivery efficiency to the posterior eye segment. A mouse model was established to explore the delivery efficiency of GEH in +/− surface charge using two methods: eye drops and via subconjunctival injection (see Figure 1b). The whole eyeball cryosection with fluorescence was examined and quantified so as to observe its transportation in the eyes using these two methods.

## 2. Materials and Methods

### 2.1. Reagent and Chemicals 

Gelatin type A (bloom 110 from porcine skin), EGCG (≥95%), cell counting kit 8 (CCK-8), live/dead cell double-staining kit, and fluorescent aqueous mounting medium were acquired from Sigma-Aldrich (St. Louis, MO, USA). HA was purchased from Maxigen Biotech Inc. (ArtiAid^®^ Intra-articular injection, New Taipei City, Taiwan). Mixed 5(6)-carboxy-tetramethylrhodamine succinimidyl ester isomers (TAMRA-ES), penicillin–streptomycin–neomycin (PSN), and 4′,6-diamidino-2 phenylindole (DAPI) were acquired from Thermo Fisher Scientific (Waltham, MA, USA). A Vivaspin 500 ultrafiltration device (molecular weight: 30 kDa) was purchased from Sartorius (Göttingen, Germany). Phosphatase buffer saline (PBS), trypsin–EDTA, fetal bovine serum (FBS), and Dulbecco’s Modified Eagle Medium/Nutrient Mixture F-12 (D-MEM/F-12) were obtained from Life Technologies (Eugene, OR, USA). Zoletil 50 solution was acquired from Virbac Animal Health (Carros, France), and 2% Rompun was purchased from Bayer (Leverkusen, Germany). Topical anesthesia solution (Alcaine^®^ 0.5%) was purchased from Alcon (Hünenberg, Switzerland). 0.22 µm Spring filter (Acrodisc 25 mm w/0.2 µm Supor STRL) was purchased from Pall Corporation (Port Washington, NY, USA). The remaining chemicals were purchased from Sigma-Aldrich.

### 2.2. Preparation and Characterization of GE NPs with/without HA Coating

The method for synthesizing GE NPs was used as per our previous study, albeit with slight modifications [22]. Firstly, gelatin powder was dissolved in warm-distilled H_2_O (45 °C), which was stirred to get a gelatin solution (4.5 mg/mL); afterwards, 450 µL) EGCG (4.5 mg/mL aqueous solution) was quickly added to the gelatin solution (450 µL) and stirred at 990 rpm to form NPs as a colloidal solution at room temperature. The NPs with gelatin/EGCG were named GE-NPs based on the self-assembly process (Figure 1a). After the GE-NPs’ preparation, HA (100 µL) was added to the GE-NP solution finally at different HA concentrations (62.5 µg/mL and 167 µg/mL). GE-NPs could be synthesized with positive (GEH+) or negative (GEH−) surface charges, and were continuously stirred for 10 min. A schematic drawing of the two types of GEH−NPs is shown in Figure 1a. All raw material solutions (gelatin, EGCG) were sterilized via a 0.22 µm filter, HA being already a sterilized product. Then the NPs’ synthesis protocols were performed in a laminar flow. The sizes and zeta potentials of the NPs were examined using dynamic light scattering (DLS) (Zetasizer Nano ZS90, Malvern Instruments, Malvern, UK). The morphology of the NPs and their appearance after HA coating were observed using transmission electron microscopy (TEM, HT7700, Hitachi, Tokyo, Japan). The NPs were diluted, dropped on a nickel mesh, and then dried for examination by TEM.

### 2.3. In-Vitro Test

#### 2.3.1. Cytotoxicity Evaluation

Adult human retinal pigment epithelial cells (ARPE-19) were purchased from the Bioresource Collection and Research Center (BCRC, Hsinchu, Taiwan) and grown in a DMEM/F12 medium containing 10% FBS and PSN. ARPE-19 cells (5000 cells/well) were seeded into 96-well plates overnight and then co-cultured with different tested samples. Equal volumes (200 µL) of EGCG, GE-NPs, GEH+ and GEH− NPs in various EGCG concentrations (0.2~50 µg/mL) were tested for co-culturing with ARPE-19. After incubation for 1 d at 37 °C, the cell viability was examined via CCK-8 kit using the WST-8 (2-(2-methoxy-4-nitrophenyl)-3-(4-nitrophenyl)-5-(2,4-disulfophenyl)-2H-tetrazolium) reduction assay procedure as instructed. After 3 h of incubation with the WST-8 reagent, the medium was examined at an absorbance wavelength of 450 nm using a microplate reader (Varioskan^®^ Flash Spectral Multimode Readers, Thermo Scientific, Waltham, MA, USA).

#### 2.3.2. Viable Cells Staining

The cell viability of various NPs was examined using the live/dead cell double staining kit, for both fluorescence staining of live (green) and dead (read) cells. ARPE-19 cells were seeded into the 24 wells at a concentration of 5 × 10^4^ (cells/well) and cultured overnight. The tested groups were as follows: positive control (culture medium), EGCG solution, GE, GEH+, and GEH− NPs at EGCG concentration of 20 μg/mL. The cells were co-cultured with the test group for 1 d. The negative control group was treated with 1% Triton X-100 prior to staining. The calcein-AM agent was used, and the acetoxymethyl ester of calcein had high cell membrane permeability, was lipophilic, and could stain live cells in green fluorescence. The cells were then examined under a fluorescent microscope (Leica, DMi8, Wetzlar, Germany), and the fluorescence intensities of the images were quantified using ImageJ software (http://imagej.nih.gov/ij/ accessed on 9 January 2017; provided in the public domain by the National Institutes of Health, Bethesda, MD, USA). 

#### 2.3.3. Cellular Uptake of NPs

Flow cytometry was used to examine the intracellular signal of NPs to determine their uptake in ARPE-19 cells. All NPs (GE, GEH+, GEH−) were conjugated with a fluorescent dye (TAMRA-ES). The ARPE-19 cells (1 × 10^5^ cells) were seeded in a 6-well plate with a culture medium overnight and then incubated with various fluorescent NPs at 37 °C for 1 h. Only the TAMRA solution was tested as a free dye. After culturing with ARPE-19 for 1 h, the cells were detached using trypsin-EDTA to obtain a single-cell suspension. The cells were then examined using a flow cytometer (FACSCanto II, BD Biosciences, Franklin Lakes, NJ, USA) at Ex/Em: 546 nm/579 nm. To confirm the effect of HA binding to the CD44 receptor on ARPE-19 cells, cells were pretreated with HA (50 μg/mL) for 30 min, then co-cultured with nanoparticles and examined as described above. 

### 2.4. In-Vivo Test

Wide-type C57BL/6J black mice, aged 8–14 weeks, were used for the in vivo test. The experimental procedure was approved by the Institutional Animal Care and Use Committee (IACUC) of the Taipei Medical University (No. LAC-2017-0344). To anesthetize the mice, an intraperitoneal injection of a combination solution of Zoletil (tiletamine/zolazepam) and Rompun (xylazine) was prepared and injected into mice at a final concentration of Zoletil (20 mg/kg) and xylazine (10 mg/kg). One drop of topical anesthesia agent, Alcaine^®^ (oxybuprocaine hydrochloride 0.5%), was applied to the mouse eye.

#### 2.4.1. Topical Administration—Eye Drops

Eye drops with dye-labeled NPs were tested, and all formulations (GE, GEH+ GEH− NPs, and free dye) were diluted with PBS to adjust them to the same fluorescent concentration and sterilized by go through a 0.22 μm filter. After the mice were anesthetized, eye drops (5 μL) were instilled twice on the lower part of the eye surface, followed by a 30 min waiting period. Subsequently, the mice were sacrificed and the whole eyeball was removed, washed, immersed in 4% paraformaldehyde (PFA), stored at 4 °C refrigerator for tissue fixation, and readied for cryosectioning.

#### 2.4.2. Subconjunctival Injection (SCI)

An insulin syringe with a 30 G needle was used for subconjunctival injection. Free fluorescent dye and various fluorescent NPs (GE, GEH+, GEH−) were injected into the subconjunctival area (10 μL) of the mouse eye individually under a stereo dissecting microscope (SAGE vision, SL-730, New Taipei City, Taiwan). The injection site was located at the junction between the iris and conjunctiva, and the needle was placed in the horizontal direction at an angle of 10 to 15 degrees to the ocular surface. Successful injection of a blister into the conjunctiva bulge was observed. Thirty minutes after injection, the mice were sacrificed, and the whole eyeballs were removed, washed with PBS, and then immersed in 4% PFA for cryosection.

#### 2.4.3. Cryosection Examination via Slide-Based Tissue Cytometry

The fixed eyeballs were embedded in optimal cutting temperature compound for cryosection; slices were sectioned in 10 μm thickness by a cryostat microtome (CM 3505S, Leica, Germany) at −20 °C, then attached to the positively charged glass slides (Superfrost^®^ Plus, Thermo). DAPI solution (300 nM) was used for nuclear staining, and the slides were mounted. Slide-based tissue cytometry, Tissue FAXS (TissueGnostics, Vienna, Austria), was used to capture whole eyeball images. Finally, the red fluorescent area in the posterior segment was quantified using the ImageJ software. The fluorescent area was calculated by dividing the red fluorescent area by the selected ocular area, which included the conjunctiva, sclera, choroid, and retina.

$$\mathrm{The}\;\mathrm{fluorescent}\;\mathrm{area}\;\left(\%\right)=\frac{\mathrm{Red}\;\mathrm{fluorescent}\;\mathrm{area}}{\;\mathrm{Selected}\;\mathrm{ocular}\;\mathrm{tissue}\;\mathrm{area}}\times 100$$

### 2.5. Statistical Analysis and Visualizations

All experimental results were reported as mean ± standard deviation (SD). Statistical analyses were performed using the Excel software in two-tailed paired *t*-tests. *p*-Values less than 0.05 were considered significant differences in mean test variables (* *p* < 0.05). 

## 3. Results

### 3.1. Characterization of NPs

**Figure 2:**
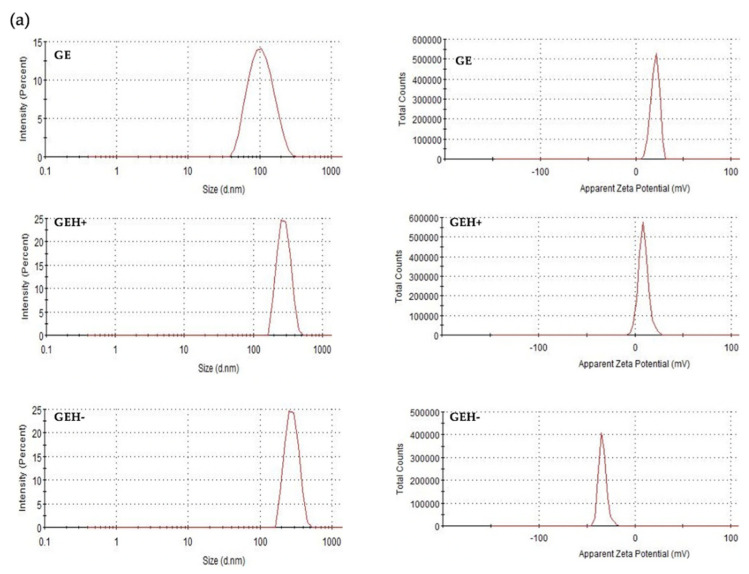

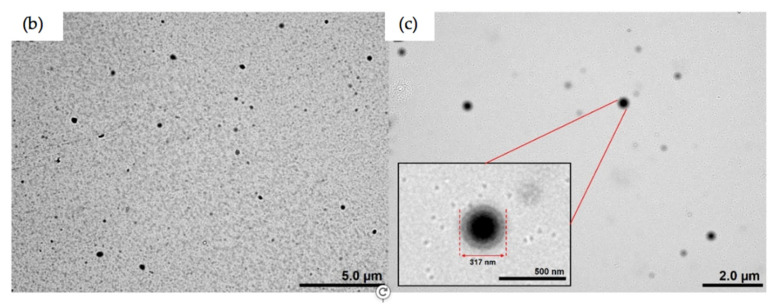
DLS results of size/zeta potential pattern of (**a**) GE, GEH+, and GEH− NPs; and photos of TEM acquired from (**b**) GE and (**c**) GEH− NPs.

The size and surface charge (zeta potential) were determined via DLS (data shown in Table 1). The GE-NPs were positively charged (20.4 mV), with a size of ~100 nm. With HA addition, both GEH+ (273.20 ± 3.22 nm) and GEH− (353.50 ± 28.54 nm) became larger than the GEs. With different amounts of HA addition, the zeta potential of the 62.5 μg/mL HA addition is +9.16 mV (GEH+), and −35.4 mV for the HA addition at 167 μg/mL (GEH−). Larger particle size and more negative charged NPs were observed. The polydispersity index (PdI) of all NPs was <0.4, confirming that the NPs were well suspended. The size distribution and zeta potential pattern in a single peak of GE, GEH+, and GEH- NPs were observed (Figure 2a). The morphology of both positive NPs (GE) and negative NPs (GEH−) were round spheres with no aggregation (Figure 2b,c). The shape of GEH+ NPs was similar to that of the other two (not shown here). In addition, a shell layer was observed in GEH−, and it is speculated that the HA coating appeared on the surface.

### 3.2. Cytotoxicity and Cell Viability

**Figure 3:**
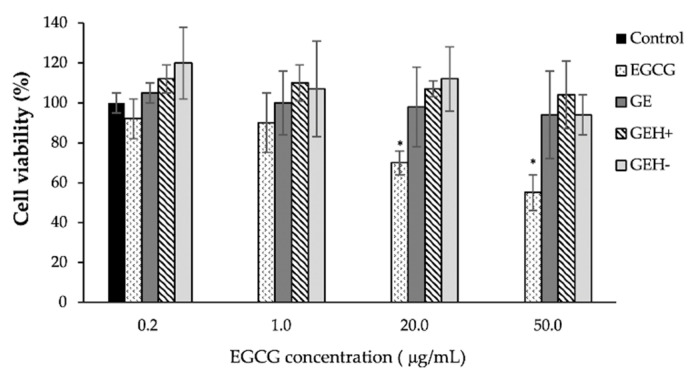
Cell viability of ARPE-19 cultured with variant NPs formulation in different EGCG concentrations for one day (mean ± SD, n = 6), * *p* < 0.05 compared with control.

The cytotoxicity of the NPs was tested using the CCK-8 kit. Given that these are self-assembled NPs with EGCG, the appropriate EGCG concentration for ARPE-19 needs to be confirmed. In Figure 3, non-toxicity was observed when treated by EGCG contained NPs (GE, GEH+, GEH−) at all tested EGCG concentration levels (0.2~50 μg/mL), with cell viabilities presenting at >95%. Lower cell viability was observed in the EGCG group at concentrations >20 μg/mL (70–55%, * *p* < 0.05) and may be due to its quick effects on cells.

**Figure 4:**
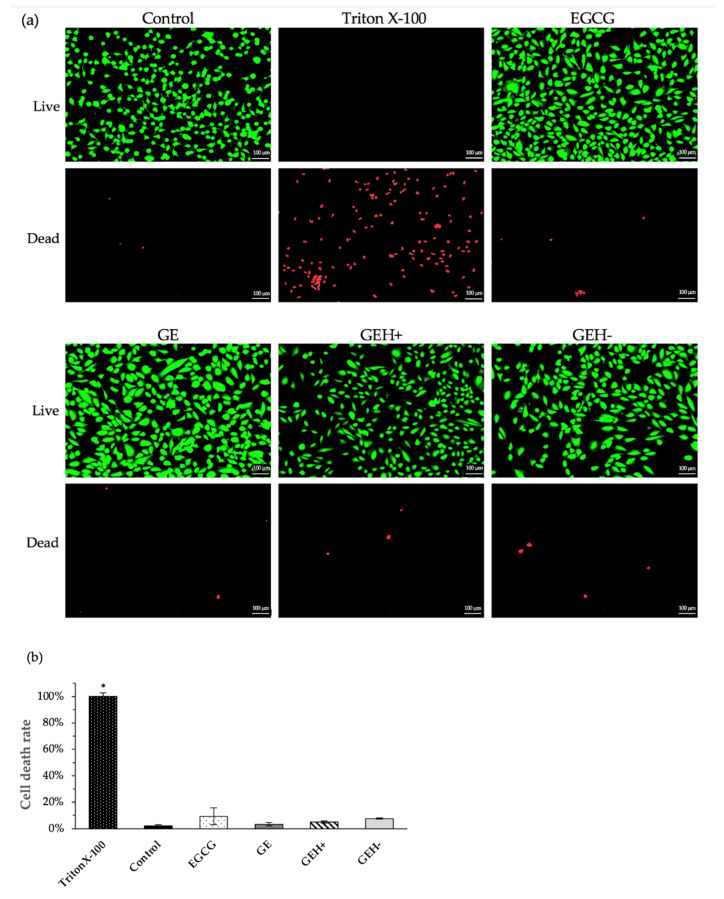
(**a**) Photos of live/dead cells staining of ARPE-19 cells when cultured with variant NP formulation at EGCG concentration of 20 µg/mL for one day (scale bar: 100 µm). (**b**) Image’s quantification of cell death rate (mean ± SD, n = 3), * *p* < 0.05 compared with control).

Representative images of the live/dead cells stained with fluorescence are shown in Figure 4a. Many green live cells were observed after day 1 of cultivation with a variant formula, except for Triton X-100. A few red spots were observed in the control, EGCG, GE, GEH+, and GEH− NP groups. Many dead cells (red spots) were observed in the negative control (Triton X-100).

The quantified data for counting dead cells from three photographs of each treatment condition are shown in Figure 4b. The rate of cell death was calculated by dividing the number of dead cells by the number of dead Triton X-100 cells. The death rate of the control group was 2.1% ± 0.9%, representing natural culture conditions. The death rate of EGCG (20 μg/mL) was slightly higher (9.3% ± 6.4%), which agrees with the CCK-8 result. The death rates of GE, GEH+, and GEH− at the same EGCG concentration (20 μg/mL) were 3.4% ± 2.1%, 5.1% ± 0.9%, and 7.6% ± 0.4%, respectively. None of the tested agents caused ARPE-19 cell death.

### 3.3. GEH+ NPs Highly Uptake 

**Figure 5:**
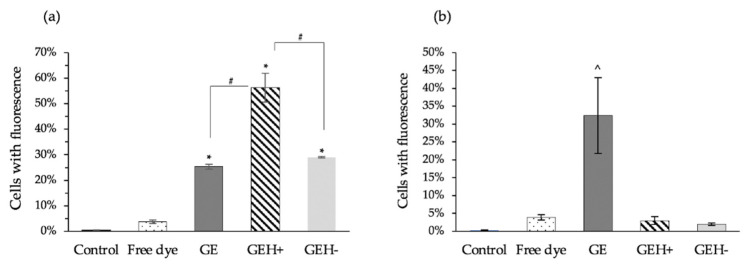
Fluorescent cells percentage examined by flow cytometry: (**a**) 1 h incubation with variant formula, and (**b**) pretreated with HA, then cocultured with dye containing NPs. (mean ± SD, n = 3) (* *p* < 0.05 compared with free dye, # *p* < 0.05 compared with GEH+, ^ *p* < 0.05 compared with all groups).

The ARPE-19 cellular uptake was examined using flow cytometry by detecting the red fluorescent signal from dye (TAMRA)-conjugated NPs. All cells treated with NPs (GE, GEH+, GEH−) had a higher fluorescent signal (25–57%) than those treated with free dye (4.14%) after 1 h incubation, with fluorescence rates amounting to 25.3% (GE), 56.2% (GEH+), and 29.0% (GEH−) (Figure 5a). This was much higher than that of the free dye group (3.7%), indicating that these NPs could enhance dye/drug delivery into cytoplasm. Both GE and GEH+ had a positively charged surface, and GEH+ had a higher uptake rate than GE, which may contribute to the decoration of HA on the NPs’ surface. GEH+ and GEH− NPs all had an HA coating, but the opposite surface charge resulted in different uptake rates (GEH+ NPs: 56.2% ± 5.6% vs. GEH− NPs: 29.0% ± 0.3%). The uptake efficiencies of GE and GEH − were similar. To confirm the effect of HA binding to the CD44 receptor in ARPE-19 cells, a blocking test (pretreated with HA) was performed, as shown in Figure 5b. The fluorescence-containing cells in the GEH+/GEH− NPs were both reduced to <5% in the HA competition test, showing that GEH+/GEH− NPs enter cells via HA-CD44 mediated endocytosis. But the GE treated one was non-affected by the HA addition, with an uptake rate of 32.4% ± 10.6%, which is significantly higher than other groups (^ *p* < 0.05).

### 3.4. NPs’ Distribution in Eyes

**Figure 6:**
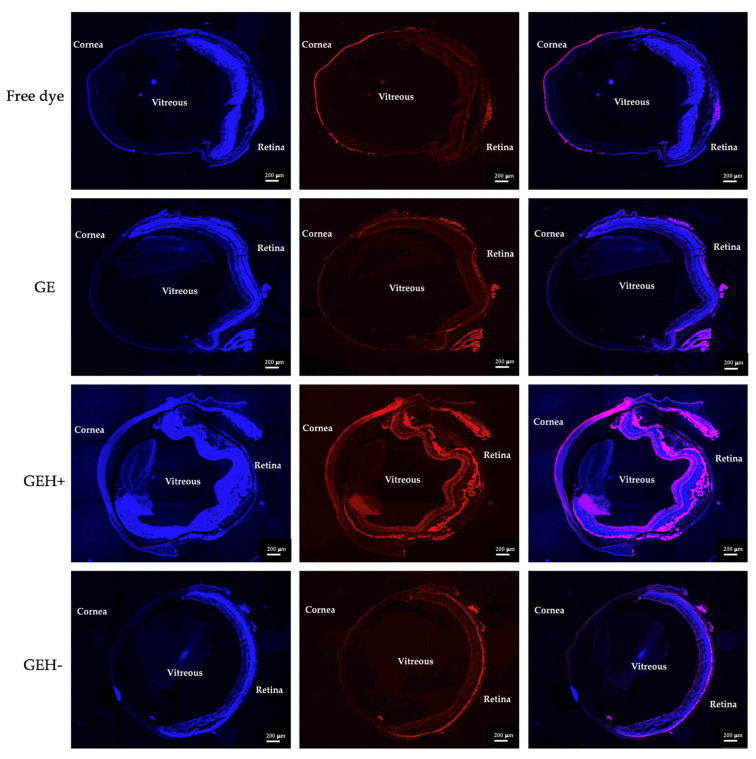
Fluorescent images of whole eyeball sections treated by different nanoparticles via topical administration (eye drops), after dosing for 30 min. (scale bar: 200 µm) (Nuclear stained by DAPI in blue; dye (TAMRA)-conjugated NPs revealed in red; and the merged images).

We compared various formulations delivered by two different administration methods (eye drops and subconjunctival injection, Figure 1b) to the mouse eye to observe dye-conjugated NPs distributed throughout the eyeball. Images of the eyeball treated with variant NPs by topical administration (eye drops) after 30 min are shown in Figure 6. A red fluorescent signal was observed in the cornea of the free dye-treated group, and almost no red fluorescence was observed in the retina. This means that the free dye (drug) solution was difficult to deliver and penetrate the eye, making it difficult to arrive at the ocular posterior segment (retina). The red fluorescence intensity in GE, GEH+, and GEH− NP-treated cells at the posterior area (retina) was much higher than that of the free dye group. The red fluorescence in the cornea can still be observed in the GEH+ NP-treated eye, but it was observed that GEH+ NPs not only existed at the RPE outer surface (choroid) but also inside the retina. The fluorescence intensity of GEH+ in the posterior segment was higher than that in the free dye and GE NP groups, and in the GEH− NP-treated eye, the red fluorescence in the posterior region was stronger than that in the cornea. This revealed that HA coating on the NP surface can help NPs to be delivered to the retina. Although the size of GEH+ NPs was larger than that of GE NPs, the results indicated that surface modification with HA was helpful for posterior eye drug delivery.

**Figure 7:**
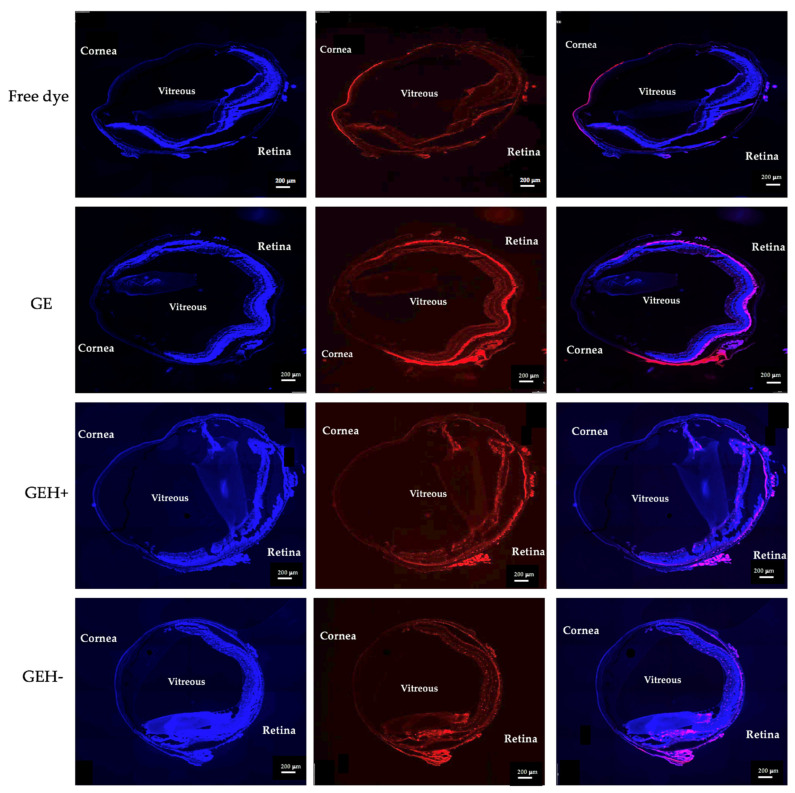
Fluorescent images of whole eyeball sections treated by different nanoparticles via subconjunctival injection, 30 min after injection. (scale bar: 200 µm) (Nuclear stained by DAPI in blue; dye (TAMRA)-conjugated NPs revealed in red; and the merged images).

The current drug administrated to the posterior segment still focuses on injection methods (compared with intravitreal injection); a less invasive injection, subconjunctival injection (SCI), was selected here. Free dye was only observed in the cornea and conjunctiva and was not found in the posterior site (Figure 7). In the GE NP-treated eyes, strong fluorescence surrounding the outer posterior segment was observed in the area between the choroid and retinal pigment epithelium (RPE) layer, but not inside the retina. In the GEH+ -treated group, NPs with strong fluorescence were not only found in the RPE layers, but also in the inner retina (Figure 7). The distribution of GEH− NPs was similar to that of GEH+ NPs via the SCI method, but weaker fluorescent intensity and a lesser amount was found in the retina, probably due to the larger size which cannot be effectively transported to the posterior segment during travel from the conjunctiva to the choroidal and then to the retina via sclera pores.

**Figure 8:**
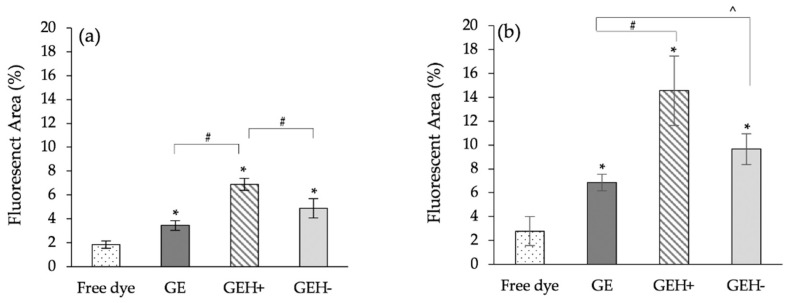
Quantification of posterior eye segment with red fluorescence: (**a**) eye drops, and (**b**) SCI treatment. Mean ± SD, n = 9. (* *p* < 0.05 compared with free dye, ^ *p* < 0.05 compared with GE, # *p* < 0.05 compared with GEH+).

The fluorescence quantification of cryosections is shown in Figure 8. In the topical delivery method (eye drops), the red fluorescent area in the posterior eye region from the free dye-treated group was 1.9% ± 0.3%, the GE group was 3.5% ± 0.4%, the GEH+ group was 6.9% ± 0.5%, and the GEH− group was 4.8% ± 0.8% (Figure 8a). All groups treated with dye-conjugated NPs had a higher signal than the free dye-treated group. Moreover, the HA-modified groups (GEH+/GEH−) had a higher fluorescent area percentage than the non-modified groups, although the particle size of GE was smaller than that of GEH+ and GEH− NPs. The surface charge and ligand-modification effects are more effective than the size effect. The GEH+ NPs-treated eyes had the largest fluorescent area in the ocular posterior segment for eye drops, which was significantly higher than that of GE and GEH− NPs (# *p* < 0.05, Figure 8a). The fluorescent area percentage in the eyes treated via subconjunctival injection (SCI) is shown in Figure 8b. The area of the free dye group was 2.79% ± 1.2%, which is much lower than that of the nanoparticle groups. The area of GE, GEH+, and GEH− is 6.86% ± 0.7%, 14.55% ± 2.9%, and 9.66% ± 1.3%, respectively. The posterior eye delivery efficiency via SCI is higher than topical administration (eye drops), but the tendency of the fluorescent accumulation ratio between variant NP formula is similar in two different administrative ways. Regardless of the delivery by topical administration (eye drops) or SCI, the delivery efficiency of NPs is better than that of the free dye (drug) solution. In addition, NPs with a positive charge and with an HA modified (GEH+) surface can enhance the ocular posterior delivery efficiency (^ *p* < 0.05, compared with GE). The GEH+ NP-treated eyes had a higher fluorescent signal than GEH− NPs, although the difference was not significant. The lower amount of GEH− NPs in the posterior eyes may be due to the larger size (~350 nm), which cannot move to the posterior segment.

**Figure 9:**
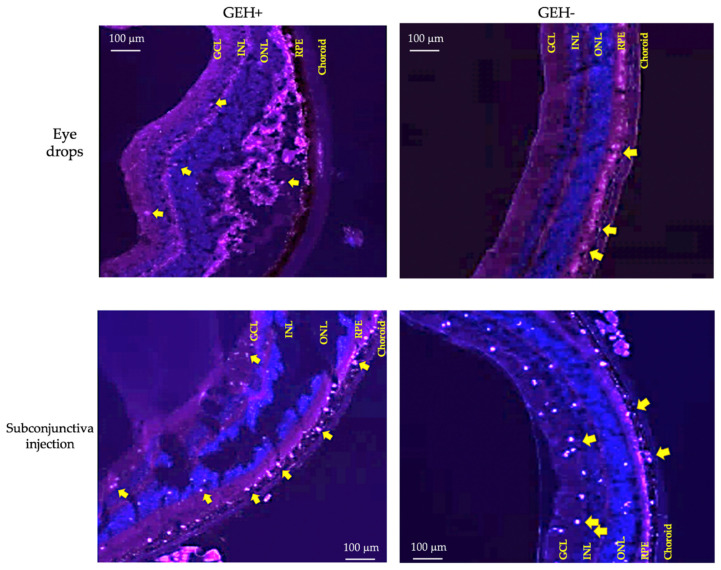
Photos of fluorescent NP (yellow arrow) distribution in the stratified retina after topical administration and subconjunctival injection after 30 min dosing. (GCL: ganglion cell layer, INL: inner nuclear layer, ONL: outer nuclear layer, RPE: retinal pigment epithelial layer).

From Figure 6 and Figure 7, the posterior eye images of the GE group revealed that these NPs were extremely present in the choroid, and very low red fluorescent spots were found in the RPE layers. GE NPs were blocked in the RPE layer owing to RPE-tight junction barriers, even though their size was the smallest (98 nm). HA-modified NPs (GEH+/GEH−) were taken up by RPE cells via HA-CD44 receptor-mediated endocytosis. In Figure 9, red spots representing GEH+ NPs and GEH− NPs localized in the retinal layers were observed in both administrative ways. In the GEH+ NPs treated group, the fluorescent dots were mainly accumulated in the RPE layers, but some red dots were still found in the internal layers, such as the inner nuclear layer (INL) and the ganglion cell layer (GCL) for both delivery methods. In the GEH− treated groups, the red dots of GEH− mainly accumulated in the RPE layer, which may be due to the larger size and endocytosis by RPE cells. Surprisingly, the SCI delivered one (Figure 9), many red dots were found in the inner layer, including the outer nuclear layer (ONL), inner nuclear layer (INL), and ganglion cell layer (GCL), in the order from retina to vitreous.

## 4. Discussion

For the posterior eye—such as the retina and choroid—intravitreal injection (IVT) is the main method used to deliver drugs or therapeutic agents to the area. Complications such as endophthalmitis, hemorrhage, and retinal detachment usually occur after IVT [27]. Topical delivery of eye drops is the first choice for eye disease treatment if the patient is given the option. However, less than 0.1% of topical therapeutic agents can be delivered to the retina [28]. Nowadays, nanomedicine can be used to improve drug delivery efficiency to the posterior of the eye. One advantage of nanoparticles/nanomedicine for drug delivery is that these nanocargoes can enter cells via endocytosis, facilitating nanoparticle and drug content in the cytoplasm, and preventing drug release or deactivation before reaching cells [29]. Moreover, nanoparticles can help prevent tear washout and reduce nasolacrimal clearance by providing a higher drug concentration in ocular tissue with a sustained release of drugs [9,30,31]. Nanoparticle distribution in the eye is influenced by many factors, including composition, size, charge, surface ligands, and administration route. Therefore, the design of nanoparticles and the administration route must be considered when designing an ocular nanoparticle system-based anterior/posterior application.

The gelatin/EGCG self-assembling nanoparticles were synthesized according to our previous study [22], replicated here by controlling the HA-added amount to form cationic/ionic nanoparticles with the size <400 nm (Table 1, Figure 2). These NPs were used to study how NP properties influence their distribution in eyes by different delivery methods. Originally, the size and charge of GE NPs were 98 nm and 20 mV, respectively. With some HA addition (negatively charged carboxylic group (−COOH)), the GEH+ NPs still possessed a positively charged surface (9.16 mV). With high amount (167 μg/mL) of HA addition, its overcome bioelectrical condition became a negative surface (GEH− NPs, −35 mV). With more HA addition, the particle size increased (98 nm → 273 nm → 353 nm). Originally, GE NPs were positively charged (20 mV). At a low concentration (62.5 μg/mL) of HA, the amount of HA with COO− was not high enough to neutralize the (+) GE NPs, so the overall charge with HA-coated GE NPs remained in the positive condition (GEH+, 9 mV). With a higher concentration of HA, the content of COO- in HA was higher than that in GE NPs(+); therefore, its zeta potential was negative (GEH−, −35 mV).

Since EGCG is a polyphenol with anti-inflammatory and antioxidant capacities, the tolerable concentration for treating ARPE-19 cells needs to be confirmed. Based on the results of the cell viability test and live/dead staining examination, we confirmed that all GE-based NPs at EGCG concentrations <50 μg/mL are non-toxic to ARPE cells (Figure 3 and Figure 4). Finally, all types of NPs (GE, GEH+, and GEH−) with EGCG at 20 μg/mL complex were chosen for in vivo ocular drug delivery. In addition, the uptake of NPs by ARPE-19 cells was evaluated by flow cytometry. It can be found that the intracellular fluorescent signal in the NP groups (GE, GEH+, GEH−) was higher than that of the free dye group (Figure 5a), which reaffirms that NPs as drug carriers can help effectively transport drugs into cells. The cell membrane potential was −30 to ~−80 mV, so GE (20 mV) and GEH+ (9 mV) NPs could have stronger charged interactions with the cell membrane, increasing the amount of NP absorption on the cell and getting into the cytoplasm. CD44 is a cell surface receptor for HA and it is widely expressed in the RPE of retina [23]. In some retinal diseases, the CD44 on RPE cells could greatly overexpress in the diseased/inflamed eye [32,33]. Therefore, HA-modified NPs can specially target to normal and inflamed RPE cells. Huang et al. revealed that HA-coated human serum albumin (HSA) NPs exhibited enhanced in vitro cellular uptake and ex vivo retinal penetration via HA-CD44 receptor-mediated interactions [23]. A similar result was found for the intracellular accumulation of ARPE-19 cells (Figure 5a) with HA-CD44 mediated endocytosis (Figure 5b). GEH+ NPs with cationic and HA modification properties were absorbed on the cell membrane and also proceeded via receptor-mediated endocytosis; the highest uptake amount in ARPE-19 cells was observed here. However, the GE NPs (positive charge) and GEH− (HA modification) possessed only one of the advantageous opportunities for enhancing cellular uptake efficiency, so the amount of these two NPs in cells was considered to be similar (Figure 5a). 

For the in-vivo test, two administrative methods (topical administration, via eye drops; and subconjunctival injection, SCI) were adopted, and the results of each delivered method were evaluated by whole eyeball cryosections with DAPI staining to label the nuclei, allowing for NPs with red fluorescence to be revealed (Figure 6, Figure 7, Figure 8 and Figure 9). The content of NPs (fluorescent area) in the SCI group that accumulated in the posterior eye was higher than that in the topical demonstration group. Topical delivery is an efficient administration method for the treatment of anterior (cornea/conjunctiva) diseases. However, multiple ocular barriers—such as tear drainage, cornea, conjunctiva, and the blood-aqueous barrier—are inefficient formulas for posterior eye treatment. Nevertheless, eye drops are more convenient and friendly to patients than other methods, especially intravitreal injection (IVT) [34]. Researchers have focused on the development of topical formulations for the treatment of retinal diseases. Lacrimal draining is one of the routes for drug delivery from the cornea to the retina via the circulatory system; however, it can be neglected because of the trace amount of drug forward remaining via this way due to the blood-retina barrier (BRB) [9,16]. Eye drops deliver drugs to the posterior segment via three routes: (1) the trans-corneal route, (2) the periocular (trans-sclera) route, and (3) the uvea-sclera route [9,24,35]. For the trans-corneal route, the drug delivery path includes the cornea anterior chamber, passes the iris/ciliary body → vitreous humor, and finally arrives at the stratified retina (Figure 1, Route 1). The second route is the periocular (trans-sclera) route, where the drug travels to the conjunctiva and then to the posterior sclera → posterior choroid → retina. The last route, the uveal route, allows for the drug/NP to penetrate to the cornea, then anterior chamber → anterior choroid → posterior choroid, thereby reaching the retina [9,13,16,35]. The last two routes, the non-corneal paths, are quite closed, as shown in Figure 1 (Route 2). Due to the complexity of the three-dimensional network of collagen fibrils bridged by proteoglycan filaments in the vitreous body [36,37], the efficacy of eye drops delivered to the retina via the trans-corneal route (crossing over the vitreous) is significantly impaired [9,37]. It is thought that the drug reaches the retina via non-corneal routes rather than the trans-corneal route [10]. Our data confirmed this as well, given that fluorescent NPs were found in the posterior area with no fluorescence signal in the cornea and vitreous after topical delivery (eye drops, Figure 6). Positively charged NPs tend to clump in the vitreous, and anionic NPs can diffuse to the retina when they travel in the vitreous [9,28]. Since the major route for NP delivery to the retina via eye drops does not pass through the vitreous, the charge effect does not play a major role. Tahara et al. synthesized chitosan-coated poly (D,L-lactide-co-glycolide) (PLGA) NPs with a size of 330 nm and zeta potential of −9 mV for topical drug delivery to the posterior eye [24]. These fluorescent PLGA NPs were observed in the retina and reached a maximum intensity at 30 min after administration. Chitosan, a mucoadhesive polymer, improves the drug delivery efficiency to the retina. The results of this study also agree with their evidence that GEH+ NPs with mucoadhesive HA coating on the NPs’ surface highly accumulated in the retina (Figure 7 and Figure 9) via an increase in the mucus-cornea/conjunctiva interaction, thereby increasing the initial NPs content on the ocular surface and allowing it to be forwarded to the retina via the non-corneal route. Drugs/NPs can also absorb through the conjunctiva and the underlying sclera (non-corneal route) and then diffuse through scleral water channels/pores, which range from 30 to 300 nm in size [9,10]. Therefore, GEH+ NPs (273 nm) were smaller than 300 nm and could be delivered to the posterior eye through the sclera.

Compared with IVT, subconjunctival injections (SCI) are less invasive. For the drug/NPs delivered via SCI, drug/NPs can skip cornea and conjunctival barriers; therefore, they provide higher permeability to the retina/choroidal area through the sclera pores and reach the RPE layer from the choroidal vessels, which is similar to Route 2 (non-corneal route) in eye drops. The red fluorescent area of SCI delivered GEH+ NPs in the posterior eye was 2-fold greater than that in the eye drop group (Figure 8). Thus, the posterior eye delivery efficiency of SCI was higher than that of topical administration method, which has also been confirmed. In the SCI group, the fluorescent area of GEH+ NPs (14.55%) was five times higher than that of free dye (2.79%), showing the benefit of using nanoparticles for posterior ocular drug delivery.

The retinal stratifications from the choroid to the internal retina include the choroid, retinal pigment epithelium (RPE), photoreceptor layer (PRL), outer nuclear layer (ONL), outer plexiform layer (OPL), inner nuclear layer (INL), inner plexiform layer (IPL), ganglion cell layer (GCL), nerve fiber layer (NFL), and inner limiting membrane (ILM) [35,38]. When drug/NPs are delivered to the retina via the choroidal route, the NPs transported to the internal retina from the choroid may be of the order described above. The results from the histological images in Figure 6, Figure 7 and Figure 9 supported the delivery order of NPs. NPs (GEH+/GEH−) mainly accumulated in the choroid and RPE layers, and some NPs were observed in the ONL, INL, and GCL layers, which need to overcome more layers to enter the internal retina parts. A similar tendency was found in the NPs’ distribution in the retina using both the eye drops and SCI methods (Figure 9).

GEH+ NPs were the most effective nanocarriers for permeating through the epithelium to the inner parts of the retina (Figure 8 and Figure 9). This contributes to the positive charge of the NPs and the HA-targeting effect of PRE. Particularly in regard to the positively charged surface, this is one of the major advantages for GEH+ NPs to be able to deliver a greater amount to the retina because of the higher amounts of GEH+ NP adhesive on the cornea due to the positively charged attraction with the negative cell membrane of the cornea and also the mucus adhesion effect of the HA with tear mucins. The greater the amount of GEH+ NPs adhered to the cornea/conjunctiva, the higher the amount of GEH+ NPs that can be delivered to the retina via the trans-sclera and uvea-sclera routes (non-corneal path, Route 2). Although the GE NPs also possessed a positive charge and are of a smaller particle size, the amount in the posterior eye delivered by both methods was lower than that of GEH+ NPs. Therefore, surface properties and HA modification play a major role in NPs delivery to the posterior eye via topical/SCI delivery methods.

## 5. Conclusions

We successfully synthesized variant NPs for drug delivery to the posterior eye. The raw particles, GE, were ~100 nm in size and had a positive charge (20 mV). The size and zeta potential of GEH+ were 273.2 nm and +9.16 mV; and those of GEH− were 353.5 nm and −35.4 mV. The safe concentration of GEH (+/−) NPs with non-cytotoxicity for ARPE-19 cells was 20 μg/mL for EGCG from CCK-8 and live/dead staining assays. GEH+ NPs had the highest uptake rate when co-cultured with ARPE-19 cells for 1 h and also confirmed its entrance by HA-mediated endocytosis. The quantification of fluorescent signals in the posterior eyes of mice was delivered by topical administration (eye drops) or subconjunctival injection, and all types of nanoparticles (GE or GEH+/GEH−) reached the posterior eye area (choroidal/retina) more than that of the solution form (>1.5 fold). The delivery efficiency of the subconjunctival injection is better than that of topical delivery (eye drops). Overall, NPs with HA decoration on their surfaces (GEH) could lead to greater NP presence in the posterior eyes, especially in the retina. The NP surface without/with HA (GE vs. GEH) or surface charge change (GEH+ vs. GEH−) helps us to understand how to control nanoparticles for ocular drug delivery for posterior eye treatment. Overall, we applied nanoparticles as ocular drug delivery vehicles by controlling their size and surface properties so as to manipulate their distribution in the retinal area. Based on this knowledge, we can choose a suitable nanoparticle with the required properties for pharmacological management to effectively treat posterior eye disease in the future.

## Figures and Tables

**Table 1:** Size, zeta potential and PdI of NPs with/without HA addition.

	HA [μg/mL]	Particle Size [nm]	Zeta Potential [mV]	PdI
GE	0.0	98.45 ± 1.37	20.40 ± 0.46	0.170 ± 0.011
GEH+	62.5	273.20 ± 3.22	9.16 ± 0.43	0.042 ± 0.046
GEH−	167.0	353.50 ± 28.54	−35.40 ± 1.50	0.306 ± 0.033

mean ± SD (n = 5).

## Data Availability

Not applicable.
